# Supplementary figures and images for: Injecting structure-aware insights for the learning of RNA sequence representations to identify m6A modification sites
Source: PeerJ. 2025 Feb 24;13:e18878. doi: 10.7717/peerj.18878 (PMC11867033; doi:10.7717/peerj.18878)

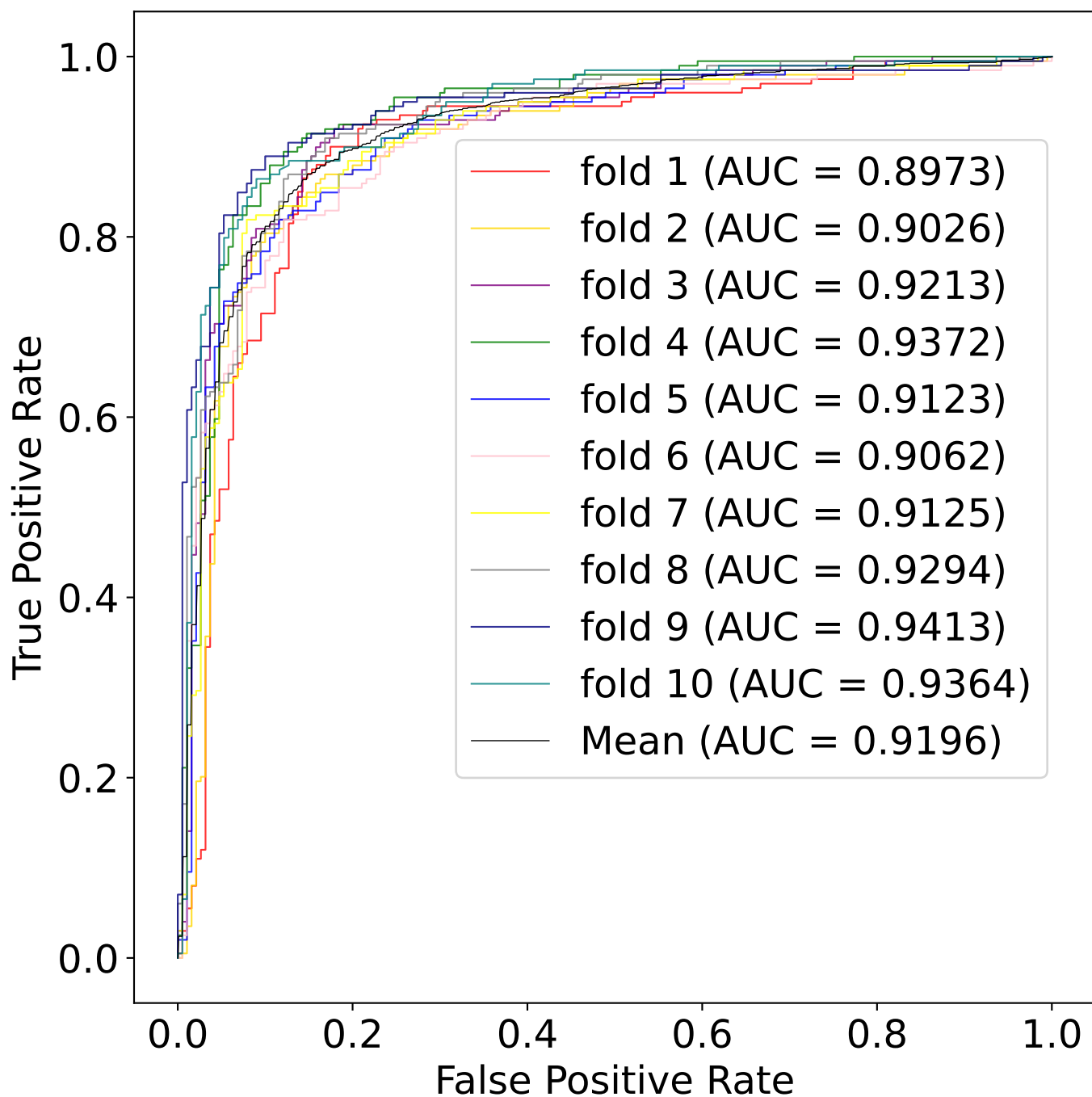

Supplement: Supplemental Information 1 [file peerj-13-18878-s001.zip › code/code/5fold-manner.pdf]

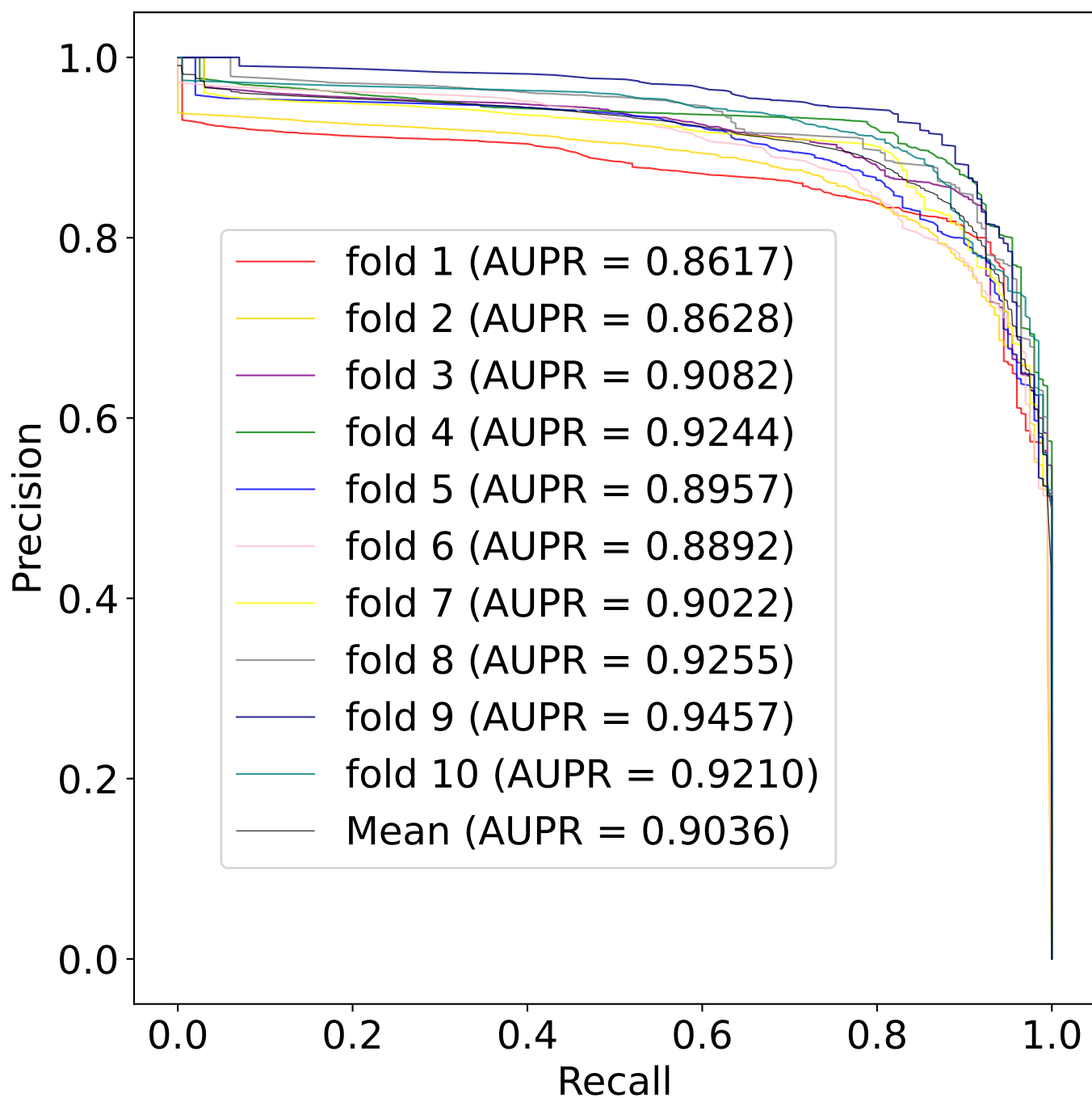

Supplement: Supplemental Information 1 [file peerj-13-18878-s001.zip › code/code/PR-5fold.pdf]
