# Supplementary material for: Injecting structure-aware insights for the learning of RNA sequence representations to identify m6A modification sites
Source: PeerJ. 2025 Feb 24;13:e18878. doi: 10.7717/peerj.18878 (PMC11867033; doi:10.7717/peerj.18878)
Supplement: Supplemental Information 2 [file peerj-13-18878-s002.docx]

Supplemental Table 1 The metrics of M6A-SAI and its variants on human-liver dataset.

| models | Acc | MCC | AUC | AUPR |
| --- | --- | --- | --- | --- |
| M6A-SAI | 0.820 | 0.640 | 0.886 | 0.874 |
| M6A-SAI-MLP | 0.481 | 0.401 | 0.494 | 0.513 |
| M6A-SAI-GCN | 0.515 | 0.477 | 0.557 | 0.526 |
