# Supplementary material for: Injecting structure-aware insights for the learning of RNA sequence representations to identify m6A modification sites
Source: PeerJ. 2025 Feb 24;13:e18878. doi: 10.7717/peerj.18878 (PMC11867033; doi:10.7717/peerj.18878)
Supplement: Supplemental Information 3 [file peerj-13-18878-s003.docx]

Supplemental Table 2 The metrics of M6A-SAI and its variants under mouse-heart dataset.

| models | Acc | MCC | AUC | AUPR |
| --- | --- | --- | --- | --- |
| M6A-SAI | 0.787 | 0.576 | 0.857 | 0.849 |
| M6A-SAI-MLP | 0.533 | 0.425 | 0.547 | 0.558 |
| M6A-SAI-GCN | 0.578 | 0.476 | 0.592 | 0.589 |
